# Phytochemical Composition and Bioactivity of *Circaea lutetiana*

**DOI:** 10.3390/molecules31122085

**Published:** 2026-06-13

**Authors:** Gaukhar Tazhkenova, Togzhan Mashan, Zhanar Iskakova, Aizhan Zeinuldina, Bakhyt Igenbayeva, Raushan Baikanova, Akmaral Kozhantayeva, Yerbolat Tashenov

**Affiliations:** 1 Department of Chemistry, Institute of Natural Sciences, L.N. Gumilyov Eurasian National University, Satpayev Street 2, Astana 010000, Kazakhstan; tazhkenova_gk@enu.kz (G.T.); zhanariskakova@mail.ru (Z.I.); tashenovyerbolat@gmail.com (Y.T.); 2 Research Institute of New Chemical Technologies, L.N. Gumilyov Eurasian National University, Satpayev Street 2, Astana 010000, Kazakhstan; 3 Department of General and Biological Chemistry, Astana Medical University, Beibitshilik Street 49A, Astana 010000, Kazakhstan; zeinuldina.a@amu.kz (A.Z.); igenbayeva.b@amu.kz (B.I.); 4 School of Medicine, Astana Medical University, Beibitshilik Street 49A, Astana 010000, Kazakhstan; baikanova.r@amu.kz

**Keywords:** *Circaea lutetiana*, phytochemistry, polyphenols, flavonoids, biological activity, antioxidant activity

## Abstract

*Circaea lutetiana* (*Onagraceae*) is a perennial medicinal species widely distributed across temperate forest ecosystems of Europe, Asia, and North America. This mini-review integrates current knowledge on the botanical characteristics, ecological distribution, phytochemical composition, and biological properties of *Circaea lutetiana*, with particular emphasis on its dominant polyphenolic constituents. Available studies demonstrate that the species is rich in flavonoids, phenolic acids, ellagic acid derivatives, and ellagitannins, among which oenothein B represents a characteristic and major constituent. Beyond polyphenols, structurally characterized glycosides, lipophilic metabolites, phytosterols, triterpenoids, fatty acids, tocopherols, and mineral elements contribute to the chemical complexity of the species. The reported biological activities of *Circaea lutetiana*, including antioxidant, anti-inflammatory, antihypertensive, and antimicrobial effects, are discussed in relation to the phytochemical profile of the plant and the biological significance of its major constituents. Recent research in green nanotechnology has additionally highlighted the potential of *Circaea lutetiana* extracts, particularly in the biosynthesis of silver nanoparticles, where plant metabolites act as reducing and stabilizing agents and may contribute to improved antimicrobial performance.

## 1. Introduction

Medicinal plants remain an important source of structurally diverse bioactive compounds with demonstrated pharmacological potential [1,2,3]. The *Onagraceae* family comprises approximately 650 species distributed across 17 genera and exhibits considerable ecological and chemical diversity [4,5,6]. Phylogenomic analysis based on 303 conserved nuclear loci has substantially improved the resolution of evolutionary relationships within *Onagraceae* and confirmed the family as an important model system for investigating floral evolution, breeding systems, and plant–pollinator interactions [7]. While several genera of this family, such as *Chamaenerion* and *Epilobium* [5], have been extensively investigated, the genus *Circaea* L. remains comparatively underexplored despite its distinct phylogenetic position [8].

**Figure 1:**
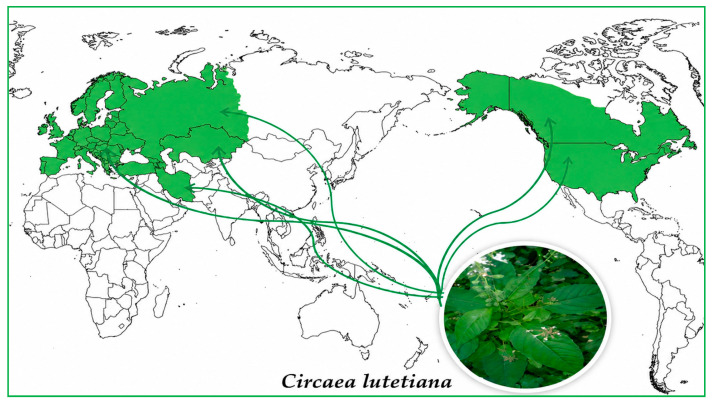
Geographic distribution of *Circaea lutetiana*.

The genus *Circaea* consists of perennial rhizomatous herbs widely distributed across temperate regions of the Northern Hemisphere [9], where they typically inhabit moist and shaded forest ecosystems. Among them, *Circaea lutetiana* is one of the most widespread species, occurring throughout Europe, Asia, and North America [9,10] with its geographic distribution presented in Figure 1. The species is characterized by its adaptation to forest understory conditions and its ecological plasticity, which contributes to its persistence across diverse habitats. In Kazakhstan, it is mainly found in forested regions such as Altai and the Dzungarian Alatau [11].

In addition to its ecological role [12], *Circaea lutetiana* has been used in traditional medicine, particularly for the treatment of wounds, inflammatory conditions, and gastrointestinal disorders [13]. Recent ethnobotanical surveys from the Caucasus region further confirm the traditional medicinal importance of *Circaea lutetiana*, documenting its continued use in local healthcare practices and highlighting its cultural significance as a medicinal forest herb [14]. Despite this traditional use, *Circaea lutetiana* remains insufficiently studied, and available data are fragmented, particularly with respect to its phytochemical composition and biological activity [15].

Available studies indicate that *Circaea lutetiana* is characterized by a high content of polyphenolic compounds, particularly flavonoids and ellagitannins, with oenothein B reported as a major constituent. These compounds have been linked to antioxidant and anti-inflammatory effects, suggesting that the species represents a promising source of biologically active metabolites [13,16,17].

Despite its wide geographical distribution and long-standing use in traditional medicine, research on *Circaea lutetiana* remains relatively limited compared with other medicinally important representatives of the family *Onagraceae*. Existing information is dispersed among taxonomic, ecological, phytochemical, pharmacological, and nanotechnology-oriented studies, making it difficult to obtain an integrated understanding of the species. Although increasing evidence indicates that *Circaea lutetiana* is a rich source of polyphenols, ellagitannins, flavonoids, and other biologically active metabolites, a comprehensive synthesis of these findings has not yet been available.

Given the growing interest in plant-derived bioactive compounds and their potential applications in pharmaceutical, biomedical, and nanotechnological fields, a critical evaluation of the available evidence is both timely and necessary. Therefore, the aim of this review is to provide an integrated overview of the botanical characteristics, ecological distribution, phytochemical composition, biological activities, and emerging nanotechnological applications of *Circaea lutetiana* while identifying current knowledge gaps, methodological limitations, and future research priorities.

## 2. Results

### 2.1. Botanical Overview and Taxonomic Position

**Figure 2:**
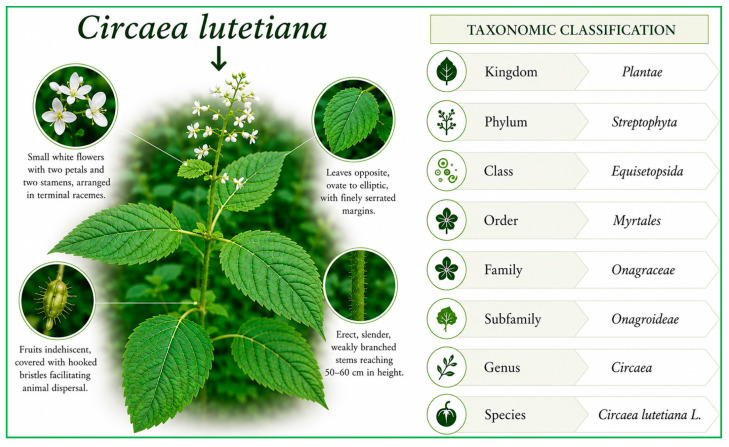
Botanical Features and Taxonomic Position of *Circaea lutetiana*.

*Circaea lutetiana* (*Onagraceae*, order *Myrtales*) is a perennial rhizomatous herb belonging to the subfamily *Onagroideae* and the genus *Circaea* (Figure 2), which comprises several species distributed across temperate regions of the Northern Hemisphere [11]. Cytogenetic investigations have demonstrated remarkable chromosomal stability within the genus *Circaea*, with most studied species and subspecies possessing a conserved chromosome number of 2*n* = 22. Such chromosomal conservatism supports the taxonomic coherence of the genus and reflects its evolutionary stability within the tribe *Circaeeae* [18]. Comparative chloroplast genomic analyses within *Onagraceae* have demonstrated a high degree of plastome conservation and suggested that *Circaea* retains a relatively ancestral plastome organization compared with several other genera of the family, supporting its evolutionary stability and distinct phylogenetic position [19].

The species is characterized by erect, slender, and weakly branched stems, typically reaching up to 50–60 cm in height. Leaves are arranged oppositely and exhibit a simple ovate to elliptic morphology with finely serrated margins [20].

Anatomical studies have shown that *Circaea lutetiana* possesses several mesomorphic characteristics associated with adaptation to moist and shaded habitats, including a thin leaf blade, bifacial mesophyll, extensive intercellular spaces, and weak development of mechanical tissues. In addition, raphide crystals have been detected throughout the roots, rhizomes, stems, and leaves, representing a distinctive anatomical feature of the species [21].

The reproductive structures are morphologically reduced but taxonomically distinctive. Flowers are small, white, and arranged in terminal racemes, with a characteristic reduction to two petals and two stamens. The inferior ovary develops into a small indehiscent fruit covered with hooked bristles, facilitating epizoochorous dispersal [22].

Overall, the simplified morphology and specialized reproductive traits of *Circaea lutetiana* reflect its adaptation to shaded forest environments [23].

### 2.2. Ecological Distribution and Forest Habitat Adaptation

*Circaea lutetiana* is a characteristic understory herb of temperate forest ecosystems and is primarily associated with mixed mesophytic and deciduous broad-leaved forests across the Northern Hemisphere [7]. The species commonly occurs in shaded woodland habitats, forest margins, and humid disturbed sites, preferring well-drained, nutrient-rich alluvial and loamy soils with sufficient moisture availability [4]. Ecological observations indicate that *Circaea lutetiana* is most frequently distributed on upper floodplains, forest terraces, and shaded slopes, whereas its occurrence in poorly drained depressions is uncommon [12].

Experimental seed-sowing studies demonstrated that *Circaea lutetiana* readily germinates and establishes in both ancient and recent forests, suggesting that dispersal limitation rather than habitat suitability may constrain its colonization of newly established woodland habitats [24].

As a forest herb, *Circaea lutetiana* demonstrates strong adaptation to local environmental conditions, particularly temperature, humidity, precipitation, and elevation. Population studies have shown that environmental heterogeneity significantly influences both morphological and genetic differentiation, suggesting ecological adaptation to forest microclimatic conditions [25]. Furthermore, reproductive performance in *Circaea lutetiana* appears to be more strongly affected by local habitat characteristics than by latitude or large-scale temperature gradients, emphasizing the importance of forest microenvironments for species persistence [26]. Additional ecological observations from Mount Lebanon further support the habitat specialization of *Circaea lutetiana* in humid forest environments. The species was reported from shaded deciduous woodland habitats occurring on humid to wet soils with thick organic litter and ferruginous sandstone substrates at elevations ranging from 1260 to 1310 m. The surrounding vegetation included mesophytic woody species such as *Fraxinus ornus*, *Platanus orientalis*, *Ostrya carpinifolia*, *Rhododendron ponticum*, and *Salix libani*, highlighting the ecological preference of *Circaea lutetiana* for cool, moisture-retaining forest microhabitats in montane ecosystems [27].

Studies on post-harvest forest dynamics revealed that *Circaea lutetiana* exhibits a marked decline following clear-cutting and shows limited recovery in recently disturbed habitats, indicating a strong dependence on stable forest microclimatic conditions [28].

### 2.3. Phytochemistry

#### 2.3.1. General Phytochemical Profile

Phytochemical investigations of *Circaea lutetiana* indicate that this species is a rich source of secondary metabolites, predominantly belonging to the polyphenolic group. The major classes of compounds reported include phenolic acids and flavonoids [13,15]. Early studies primarily relied on classical phytochemical techniques, while more recent research has applied advanced chromatographic and spectrometric methods, providing a more comprehensive characterization of its chemical composition [16].

#### 2.3.2. Polyphenolic Profile of *Circaea lutetiana*

**Figure 3:**
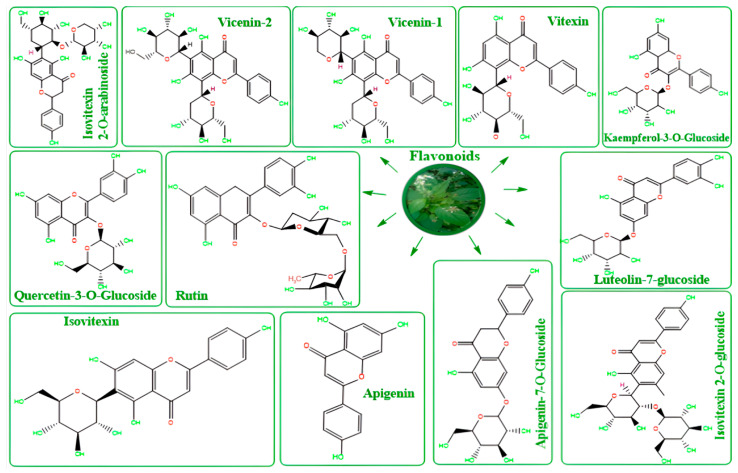
Major Flavonoids Identified in *Circaea lutetiana*.

Polyphenols represent the dominant group of bioactive constituents in *Circaea lutetiana*. Early phytochemical investigations revealed the presence of C-glycosyl flavones such as vitexin, isovitexin, and vicenin-type compounds, together with O-glycosyl flavonoids including apigenin, luteolin, quercetin, and kaempferol derivatives (Figure 3) [16]. These compounds are considered chemotaxonomic markers of the genus and highlight its distinct position within the *Onagraceae* family [29,30].

**Figure 4:**
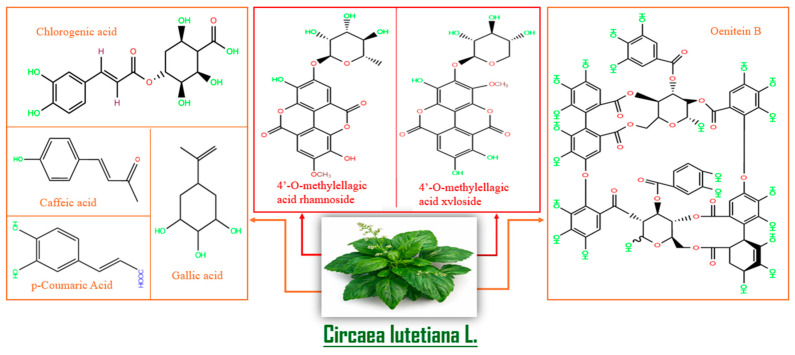
Phytochemical Constituents of *Circaea lutetiana*.

Subsequent studies employing two-dimensional paper chromatography, thin-layer chromatography, and column chromatography confirmed the diversity of flavonoids and their glycosides. Structural elucidation was achieved through hydrolysis reactions combined with comparison of chromatographic behavior and UV spectral data with reference standards [16,17]. More advanced analyses using HPLC, HPLC-UV-ESI/MS, and HPLC-DAD-MS^3^ further expanded the phytochemical profile, enabling the identification of phenolic acids, flavonoids, ellagitannins, and ellagic acid derivatives [11,13], which are summarized in Table 1. Among these, gallic acid, caffeic acid and apigenin glycosides were identified, while the macrocyclic ellagitannin oenothein B (Figure 4) was reported as a predominant constituent, confirming the tannin-rich nature of the extract [13].

In addition to flavonoids, phenolic acids and ellagitannins play a central role in defining the biological profile of *Circaea lutetiana.* Among low-molecular-weight phenolics, gallic acid represents a key structural and functional component and has been detected in different plant organs, including aerial parts [13,15]. Beyond its role as a precursor of hydrolysable tannins [31,32], gallic acid exhibits pronounced antioxidant capacity and has been associated with anti-inflammatory, antimicrobial, anticancer, and neuroprotective effects [33]. Gallic acid can be considered both a fundamental building block of polyphenolic systems and an active contributor to their biological effects [34].

Closely related to this group, caffeic acid and its derivatives further enhance the functional potential of the species. Owing to the presence of a catechol moiety, these compounds exhibit strong radical-scavenging properties and participate in the regulation of oxidative and inflammatory processes [35,36].

In contrast, high-molecular-weight ellagitannins—particularly oenothein B—represent a distinct level of biological organization [37]. This compound, identified as one of the major constituents of *Circaea lutetiana* [13,15], is recognized not only for its antioxidant properties but also for its ability to modulate cellular responses, including immune-related processes [38].

Taken together, the biological activity of *Circaea lutetiana* can be attributed to the complementary action of its polyphenolic constituents. While phenolic acids provide rapid antioxidant effects, complex ellagitannins contribute to more sustained and multifunctional biological responses [39].

#### 2.3.3. Structurally Characterized Compounds from *Circaea lutetiana*

The phytochemical investigation of *Circaea lutetiana* has resulted in the isolation of several structurally characterized secondary metabolites from the aerial parts of the plant. The compounds summarized in Table 2 represent metabolites that were isolated and structurally characterized using NMR spectroscopy and complementary spectroscopic methods, whereas Table 1 summarizes phytochemicals identified primarily through chromatographic and spectrometric profiling approaches. Kim and Kingston reported [40] the first isolation study of *Circaea lutetiana* ssp. *canadensis* using methanolic extraction of freshly collected aerial parts followed by chromatographic purification. This study led to the isolation of five compounds, including a new alkyl glycoside, 1-octyl α-d-arabinofuranosyl-(1→6)–β-d-glucopyranoside, together with isovitexin, astragalin, methyl gallate 3–O–β-d-(6′-O-galloyl)-glucopyranoside and icariside B2 (Table 2). Structural elucidation was achieved using NMR spectroscopy, HRFABMS, and acid hydrolysis.

A subsequent study by Granica and Kiss substantially expanded the number of isolated metabolites from *Circaea lutetiana*. Using hydromethanolic extraction (Methanol (aq.) 50:50, Reflux), the authors isolated sixteen compounds, mainly belonging to flavonoids, ellagic acid derivatives, phenolic acids, and ellagitannins. The isolated constituents included 3,3′,4-tri-O-methylellagic acid, gallic acid, 3-O-methylellagic acid, ellagic acid, methyl gallate, p-coumaric acid, valoneic acid dilactone methyl ester, quercetin 3-(2″-O-galloyl)-β-d-glucopyranoside, oenothein B, vicenin-1, isovitexin 2″-O-β-l-arabinoside, isovitexin 2″-O-β-d-glucopyranoside, 3-O-methylellagic acid 4′-O-β-d-xylopyranoside, 3-O-methylellagic acid 4′-O-α-l-rhamnopyranoside, isovitexin, and apigenin 6,8-di-C-α-l-arabinopyranoside. The structures of the isolated compounds were confirmed by NMR, UV–Vis, MS/MS analysis, and acidic hydrolysis. Notably, isovitexin was identified in both studies, suggesting its potential relevance as a characteristic flavonoid constituent of *Circaea lutetiana* [15,41]. Furthermore, the repeated occurrence of phenolic compounds and ellagic acid derivatives highlights the chemically diverse phytochemical profile of the species.

#### 2.3.4. Lipophilic Constituents and Non-Phenolic Metabolites

**Figure 5:**
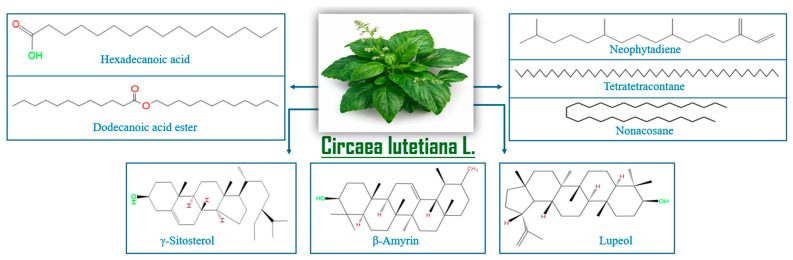
Major Lipophilic Constituents of *Circaea lutetiana*.

In addition to phenolic constituents, the phytochemical composition of *Circaea lutetiana* includes a diverse array of lipophilic and non-phenolic metabolites. The lipophilic fraction of *Circaea lutetiana* has been characterized in detail in recent studies. For example, Kozhantayeva et al. reported the composition of lipophilic constituents from the aerial parts (leaves and stems) using hexane extraction followed by GC–MS analysis [42]. A total of 25 compounds were identified, with esters (30.8%) and alkanes (12.7%) representing the predominant classes, alongside fatty acids, phytosterols, and triterpenoids. Major constituents included octadecanoic acid derivatives, 9-octadecenoic acid esters, long-chain hydrocarbons such as nonacosane and tetratetracontane, as well as γ-sitosterol, lupeol, and β-amyrin, with representative structures shown in Figure 5. The lipophilic profile of *Circaea lutetiana* is predominantly composed of lipid-derived compounds and long-chain hydrocarbons associated with plant protective functions [41], including water retention and adaptation to environmental stress [43].

Among the identified constituents, β-sitosterol is a major phytosterol with well-documented biological activity [44]. It has been reported to induce apoptosis and cell cycle arrest in cancer cells, in addition to exhibiting antioxidant and anti-inflammatory properties, supporting its significance as a bioactive component of medicinal plants [44,45].

Triterpenoids such as α- and β-amyrin represent another important group of lipophilic metabolites. These compounds have been associated with multiple pharmacological effects, including anti-inflammatory, analgesic, hepatoprotective, and antimicrobial activities, reflecting their ability to modulate diverse biochemical pathways [46,47].

#### 2.3.5. Fatty Acid and Tocopherol Profile

Seed chemistry of *Circaea lutetiana* has been investigated through the analysis of fatty acid and tocopherol composition. Velasco and Goffman reported that seed oil is dominated by linoleic (36.8%) and α-linolenic acids (28.6%), whereas γ-linolenic acid was absent. The species also exhibited a distinctive tocopherol profile characterized by elevated β-tocopherol (13.4%) and δ-tocopherol (18.0%), suggesting potential chemotaxonomic significance within *Onagraceae*. The analyses were performed using gas–liquid chromatography (GLC) for fatty acids and HPLC for tocopherol determination [48]. Fatty acid composition influences the nutritional and oxidative properties of seed oils, whereas tocopherols function as natural lipid-soluble antioxidants protecting polyunsaturated fatty acids against oxidative degradation [49,50]. Given the relatively high proportion of α-linolenic acid in *Circaea lutetiana*, its distinctive tocopherol profile may contribute to the oxidative stability of seed lipids and highlights the potential chemotaxonomic and functional relevance of this species [48].

#### 2.3.6. Mineral Composition

The mineral composition of *Circaea lutetiana* has been characterized by ICP–MS, revealing 41 macro- and microelements across different plant organs. Essential elements such as Fe, Zn, Cu, Mn, and P were identified, reflecting their role in metabolic and enzymatic processes [51].

Elemental distribution is organ-dependent, with most elements occurring within permissible limits, supporting the potential safety of the plant material. However, elevated levels of iron and beryllium in the roots indicate selective accumulation and require consideration in safety assessment.

Comparable variability has been reported in other *Onagraceae* species, where elemental composition depends on both plant organ and environmental conditions [52].

From a safety perspective, the observed organ-specific distribution of mineral elements warrants further consideration. Because elemental accumulation in medicinal plants is influenced by environmental conditions, soil composition, and the plant organ used for preparation, the higher concentrations of iron and beryllium detected in roots deserve particular attention [52]. Consequently, the safety profile of *Circaea lutetiana* cannot be assumed to be uniform across all plant parts. Although the aerial organs contained most elements within acceptable ranges, additional studies are needed to evaluate the toxicological significance of elemental accumulation in roots and to establish evidence-based recommendations for medicinal use. At present, no clinical studies have evaluated the safety of *Circaea lutetiana* preparations in humans, highlighting the need for further toxicological assessment.

#### 2.3.7. Phytochemistry–Nanotechnology Link

The phytochemical composition of *Circaea lutetiana*, particularly its richness in phenolic acids, flavonoids, and glycosylated polyphenols, provides a direct basis for its application in green nanotechnology. These metabolites possess hydroxyl and carbonyl functional groups capable of donating electrons, thereby facilitating the reduction of metal ions and stabilizing the resulting nanostructures.

In our previous study, an ethanol extract of *Circaea lutetiana*, characterized by a high content of phenolic compounds, was successfully employed for the green synthesis of silver nanoparticles (AgNPs), representing the first report of this plant as a bioreductant for nanoparticle formation. The reduction of Ag^+^ to Ag^0^ was mainly attributed to redox-active phenolic constituents, while flavonoid glycosides likely contributed to nanoparticle stabilization through surface capping [11].

Similarly, a recent study demonstrated the successful microwave-assisted synthesis of AgNPs using an aqueous leaf extract of *Circaea lutetiana*, confirming that water-soluble phytochemicals can also effectively mediate nanoparticle formation. Slight shifts in FTIR bands after synthesis suggested the involvement of hydroxyl- and carbonyl-containing metabolites in both silver ion reduction and nanoparticle stabilization [53].

### 2.4. Biological Activity

#### 2.4.1. Phytochemical Basis of the Biological Activities of *Circaea lutetiana*

**Figure 6:**
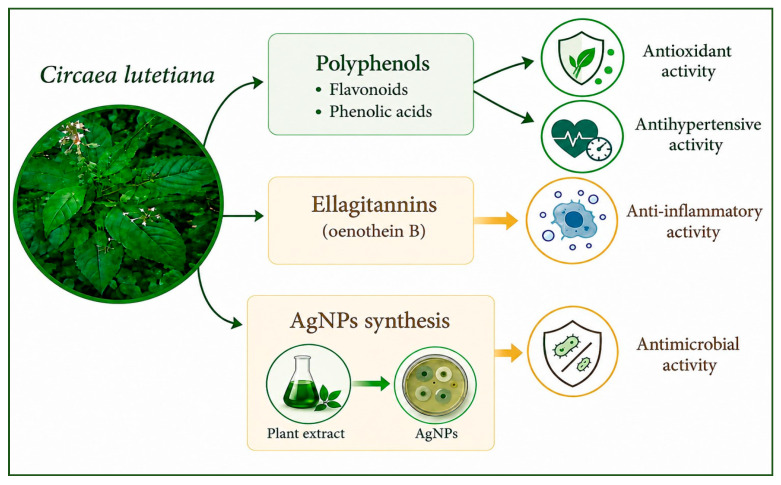
Relationship between phytochemical composition and biological activities of *Circaea lutetiana*.

The biological activity of *Circaea lutetiana* is mainly associated with its rich phytochemical composition, particularly ellagitannins, flavonoids, and phenolic acids, which are recognized as major bioactive constituents of medicinally important species within the *Onagraceae* family [11,54]. Current studies have demonstrated antioxidant, anti-inflammatory, antihypertensive, and antimicrobial properties of the species, indicating its pharmacological relevance (Figure 6).

Among the reported pharmacological properties, antioxidant activity has been the most extensively investigated. Hydromethanolic extracts of *Circaea lutetiana* demonstrated moderate scavenging activity against 2,2-diphenyl-1-picrylhydrazyl (DPPH) radicals (SC_50_ = 33.1 μg/mL), while substantially stronger activity was observed against biologically relevant reactive oxygen species, including superoxide anion (SC_50_ = 4.0 μg/mL) and hydrogen peroxide (SC_50_ < 2 μg/mL) [13]. The antioxidant effect of the extract has been primarily associated with ellagitannins, particularly oenothein B, together with other phenolic constituents identified in the plant [13,55].

Additional evidence of antioxidant potential was reported in a recent pharmacological screening of medicinal plants used for diabetes–hypertension comorbidity management. Ethanolic extract of *Circaea lutetiana* demonstrated pronounced radical scavenging activity in the ABTS assay (98.78 ± 0.75%) and exhibited strong angiotensin-converting enzyme (ACE) inhibitory activity (60.30 ± 1.72%), ranking among the most active species investigated [56]. In contrast, only weak α-amylase inhibitory activity was observed, indicating limited effectiveness in postprandial glucose regulation through this pathway [56].

Besides antioxidant activity, *Circaea lutetiana* also exhibits anti-inflammatory potential. The extract strongly inhibited hyaluronidase (IC_50_ = 13.3 μg/mL) and moderately inhibited lipoxygenase (IC_50_ = 44.7 μg/mL), indicating possible modulation of inflammation-related enzymatic pathways [13]. These activities have mainly been attributed to ellagitannin-rich fractions of the plant [13].

The antimicrobial activity of crude *Circaea lutetiana* extracts appears to be relatively limited against both Gram-positive and Gram-negative microorganisms [11]. However, recent studies demonstrated that biological activity can be enhanced through green synthesis of silver nanoparticles (AgNPs) using plant extracts. In our previous study, silver nanoparticles synthesized using the ethanolic extract of *Circaea lutetiana* exhibited significant antimicrobial activity against both Gram-positive and Gram-negative bacteria, including *Staphylococcus aureus*, *Escherichia coli*, and *Klebsiella pneumoniae* [11]. The enhanced antibacterial effect was associated with the increased biological performance of nanoscale silver combined with plant-derived phytochemicals present on the nanoparticle surface [57].

Similarly, a recent study employing an aqueous leaf extract of *Circaea lutetiana* for silver nanoparticle synthesis reported improved antimicrobial activity against *Staphylococcus aureus*, *Pseudomonas aeruginosa*, and *Candida albicans* compared to both crude plant extract and AgNO_3_ precursor solution. Antimicrobial assessment was performed using the agar well diffusion method, where biosynthesized AgNPs demonstrated larger inhibition zones against all tested microorganisms after 24 h incubation [53].

In addition to antimicrobial activity, the biosafety profile of *Circaea lutetiana* -mediated silver nanoparticles was evaluated using the WI-38 human lung fibroblast cell line. Following 48 h incubation, cell viability remained above 85% across the tested concentration range, indicating low cytotoxicity toward normal human fibroblast cells and acceptable biocompatibility of the synthesized nanoparticles [53].

#### 2.4.2. Biological Significance of Major Phenolic Constituents Identified in *Circaea lutetiana*

The phytochemical composition of *Circaea lutetiana* includes several groups of biologically active phenolic constituents, particularly phenolic acids and flavonoids (Table 1). These metabolites are widely recognized for their involvement in oxidative stress regulation, inflammatory responses, microbial inhibition, and cellular protection, making them likely contributors to the biological activities reported for the species [58]. It should be emphasized that the biological activities discussed below are derived primarily from studies of individual phytochemicals identified in *Circaea lutetiana* and do not necessarily represent biological effects experimentally demonstrated for the whole plant or its extracts. This information is presented to illustrate the potential pharmacological relevance of the major constituents detected in the species and to provide a broader context for interpreting the biological activities reported for *Circaea lutetiana* extracts.

Phenolic acids identified in *Circaea lutetiana* include gallic acid, chlorogenic acid, caffeic acid, and *p*-coumaric acid. Members of this group are well known for their antioxidant, antimicrobial, and anti-inflammatory activities and are frequently regarded as important contributors to the therapeutic value of medicinal plants [59,60]. Gallic acid, detected in the aerial parts of *Circaea lutetiana*, has been extensively investigated because of its strong radical scavenging capacity and ability to modulate oxidative stress-related pathways. Experimental studies also reported antimicrobial, anti-inflammatory, and antiproliferative activities [61].

Hydroxycinnamic acids further strengthen the biological significance of the species. Chlorogenic acid has been associated with antioxidant and anti-inflammatory effects, particularly through regulation of cellular redox balance and metabolic processes [60]. Caffeic acid demonstrated antimicrobial, anti-inflammatory, and antiproliferative activity through modulation of oxidative and inflammatory pathways [60]. Similar effects have also been reported for *p*-coumaric acid, especially regarding antioxidant protection and microbial growth inhibition [59].

Among the flavonoids identified in *Circaea lutetiana*, vicenin-1 and vicenin-2 are of particular interest because studies performed on these compounds in various experimental systems have demonstrated a broad range of biological activities. Both metabolites belong to the group of C-glycosyl flavones characterized by carbon–carbon glycosidic linkages, which confer increased chemical stability and resistance to hydrolysis. Vicenin-1 has been associated with antioxidant and anti-inflammatory activity, including modulation of oxidative stress and cytoprotective effects [62,63,64,65]. Recent studies additionally demonstrated activation of the Nrf2–ARE signaling pathway, highlighting its possible chemopreventive relevance [65]. Evidence for vicenin-2 points to anti-inflammatory, antidiabetic, and anticancer activity, including suppression of inflammatory mediators through inhibition of NF-κB signaling, interference with glycation-associated pathways, and inhibition of tumor-related signaling mechanisms [66,67,68,69].

Vitexin and isovitexin, structurally related apigenin C-glycosides identified in *Circaea lutetiana*, have been extensively investigated in different experimental models, where they exhibited antioxidant, anti-inflammatory, antimicrobial, neuroprotective, and metabolic regulatory activities [70,71,72,73]. Experimental studies indicate their involvement in the regulation of oxidative stress and inflammatory responses while increasing evidence supports their ability to modulate signaling pathways associated with apoptosis, angiogenesis, and tumor progression [70,73]. Their therapeutic relevance has additionally been explored in diabetes-related disorders through effects on insulin signaling and carbohydrate metabolism [71].

The phytochemical profile of *Circaea lutetiana* further includes several O-glycosylated flavonoids, such as apigenin-7-*O*-glucoside, luteolin-7-*O*-glucoside, rutin, quercetin-3-*O*-glucoside, and kaempferol-3-*O*-glucoside. These metabolites are widely associated with antioxidant, antimicrobial, anti-inflammatory, and anticancer properties [74,75,76]. In particular, rutin and quercetin glycosides are recognized for their strong antioxidant capacity and improved bioavailability, whereas luteolin and apigenin derivatives have been linked to modulation of inflammation- and apoptosis-related pathways [76,77,78]. Kaempferol-3-*O*-glucoside has also demonstrated anti-inflammatory and antimicrobial activity [79,80].

Beyond phenolic acids and flavonoids, phytochemical studies of *Circaea lutetiana* identified ellagic acid derivatives and the macrocyclic ellagitannin oenothein B, a characteristic constituent frequently reported in members of the *Onagraceae* family.

#### 2.4.3. Limitations and Research Gaps

Despite the growing interest in the biological properties of *Circaea lutetiana*, the currently available evidence remains limited. Most studies have been based on in vitro antioxidant, enzyme inhibition, antimicrobial, and cell-based assays, providing only an initial assessment of the biological potential of the species [11,13,56]. Although several extracts and isolated constituents demonstrated promising activity, their effectiveness under physiological conditions has not yet been verified. To date, no in vivo pharmacological investigations or clinical studies have been reported for *Circaea lutetiana*. Furthermore, information regarding the safety profile of the species is scarce and largely restricted to preliminary cytocompatibility evaluations of biosynthesized silver nanoparticles [53].

## 3. Materials and Methods

This review was conducted through a comprehensive analysis of scientific literature published on “*Circaea lutetiana*”, related species of the genus “*Circaea*”, and relevant members of the family *Onagraceae*. Literature searches were performed between January and June 2025 using the Web of Science, Scopus, PubMed, Google Scholar, and ScienceDirect databases.

The search strategy combined botanical, phytochemical, pharmacological, and nanotechnology-related keywords. The principal search terms included: “*Circaea lutetiana*”, “*Circaea lutetiana* phytochemistry”, “*Circaea lutetiana* polyphenolic compounds”, “*Circaea lutetiana* secondary metabolites”, “*Circaea lutetiana* biological activity”, “*Circaea lutetiana* antioxidant activity”, “*Circaea lutetiana* antimicrobial activity”, “*Circaea lutetiana* anti-inflammatory activity”, “*Circaea lutetiana* silver nanoparticles”, “*Circaea* and phytochemistry”, “*Circaea* and polyphenols”, “*Onagraceae*”, and “*Onagraceae* and biological activity”.

The inclusion criteria comprised peer-reviewed original research articles, review papers, and book chapters providing information on taxonomy, ecology, phytochemistry, analytical characterization, biological activity, ethnobotanical use, or nanotechnological applications of “*Circaea lutetiana*” and closely related taxa. Studies published in English were prioritized. Several classical taxonomic, cytogenetic, and phytochemical investigations published before 2000 were additionally included because they represent the primary sources describing the morphology, systematics, chromosome characteristics, and first isolation of major phytochemicals from “*Circaea*” species.

The search strategy yielded a substantial number of publications from the selected databases. After removal of duplicate records and evaluation of titles, abstracts, and full texts, studies considered most relevant to the objectives of this review were included. Particular attention was given to publications describing the taxonomy, ecology, phytochemistry, biological activity, and recent nanotechnological applications of *Circaea lutetiana*.

Due to the limited availability of studies dedicated exclusively to *Circaea lutetiana*, relevant information from other representatives of the family *Onagraceae* was considered, where appropriate, to provide additional context for the discussion of phytochemical and biological properties.

## 4. Conclusions

The available evidence indicates that *Circaea lutetiana* is a medicinal species with considerable phytochemical diversity and promising biological relevance. Studies conducted to date reveal the presence of numerous classes of metabolites, including phenolic acids, flavonoids, ellagic acid derivatives, ellagitannins, glycosides, phytosterols, triterpenoids, lipophilic compounds, fatty acids, tocopherols, and mineral elements, reflecting the chemical complexity of the species.

The biological properties reported for *Circaea lutetiana* are likely associated with the combined activity of several groups of constituents. Polyphenols, particularly flavonoids and ellagitannins, appear to be among the most important contributors, with compounds such as oenothein B, gallic acid, caffeic acid, and flavonoid glycosides potentially involved in antioxidant and anti-inflammatory effects. At the same time, lipophilic metabolites and phytosterols may provide additional biological contributions, suggesting that the pharmacological potential of the species arises from synergistic interactions among multiple metabolites.

Emerging evidence also points to new perspectives for the application of *Circaea lutetiana* in green nanotechnology. Plant-derived extracts have been successfully used in the biosynthesis of silver nanoparticles, where endogenous metabolites act as reducing and stabilizing agents and may contribute to improved antimicrobial properties and favorable biocompatibility.

## Figures and Tables

**Table 1:** Phytochemicals reported in *Circaea lutetiana* identified by chromatographic and spectrometric analyses *.

Compounds	MW	Plant Part	Identification Method	Extract Type/Method	Ref.
**Phenolic acids**					
Gallic acid	170.12	Aerial parts	HPLC, HPLC-UV-ESI/MS	Methanol (aq.), Ethanol,	[11,13]
Chlorogenic acid	354.31	Aerial parts	HPLC-UV-ESI/MS	Ethanol, Reflux	[11]
Caffeic acid	180.16	Aerial parts	HPLC-UV-ESI/MS	Ethanol, Reflux	[11]
p-Coumaric acid	164.04	Aerial parts	HPLC-UV-ESI/MS	Ethanol, Reflux	[11]
**Flavonoids**					
Vicenin-1	564.5	Aerial parts, Leaves	HPLC-DAD-MS^3^ PC, 2D-PC, TLC, UV	Methanol (aq.), Methanol, Reflux, hydrolysis	[13,16,17]
Vicenin-2	594.52	Leaves	PC	Methanol, hydrolysis	[16]
Vitexin	432.38	Leaves	PC, 2D-PC, TLC, UV	Methanol, hydrolysis	[16,17]
Isovitexin 2-O-glucoside	594.52	Aerial parts	HPLC-DAD-MS^3^	Methanol (aq.), Reflux	[13]
Isovitexin 2-O-arabinoside	564.5	Aerial parts	HPLC-DAD-MS^3^	Methanol (aq.), Reflux	[13]
Isovitexin	432.38	Aerial parts, Leaves	HPLC-DAD-MS^3^ PC, 2D-PC, TLC, UV	Methanol (aq.), Methanol, Reflux hydrolysis	[13,16,17]
Apigenin 7 O-glucoside	432.38	Aerial parts	2D-PC, TLC, UV	Methanol	[17]
Apigenin	270.24	Leaves	PC,	Methanol, hydrolysis	[16]
Luteolin- 7 O-glucoside	448.38	Leaves	PC,	Methanol	[17]
Rutin	610.52	Aerial parts	HPLC-UV-ESI/MS	Ethanol, Reflux	[11]
Quercetin-3-O-glucoside	464.38	Leaves Aerial parts	PC, UV, HPLC-UV-ESI/MS	Methanol, Ethyl acetate, Ethanol	[11,17]
Kaempferol-3-O-glucoside	432.38	Leaves	PC, UV	Methanol, hydrolysis	[17]
**Ellagic acid derivatives**					
4′-O-methylellagic acid xyloside	462.36	Aerial parts	HPLC	Methanol (aq.)	[13]
4′-O-methylellagic acid rhamnoside	462.36	Aerial parts	HPLC	Methanol (aq.)	[13]
**Tannins**					
Oenothein B	1569.1	Aerial parts	HPLC-DAD-MS^3^	Methanol (aq.)	[13]

* HPLC—High-Performance Liquid Chromatography; HPLC-DAD-MS^3^—High-Performance Liquid Chromatography coupled with Diode-Array Detection and Multistage Mass Spectrometry; HPLC-UV-ESI/MS—High-Performance Liquid Chromatography coupled with Ultraviolet Detection and Electrospray Ionization Mass Spectrometry; PC—Paper Chromatography; 2D-PC—Two-Dimensional Paper Chromatography; TLC—Thin-Layer Chromatography; UV—Ultraviolet Spectroscopy; MW—Molecular Weight.

**Table 2:** Isolated and structurally characterized compounds from *Circaea lutetiana* *.

Compounds	Classes	Extraction	Identification Method	Ref.
1-Octyl α-d-arabinofuranosyl- (1→6)-β-d-glucopyranoside	Alkyl glycoside	Methanol	HRFABMS, ^1^H-, ^13^C-NMR, DEPT, COSY, HETCOR, HMBC, TLC	[40]
Isovitexin	Flavonoid *C*-glycoside	Methanol; Methanol (aq.)	^1^H-, ^13^C-NMR, UV–Vis, MS/MS,	[15,40]
Astragalin	Flavonol glycoside	Methanol	^1^H-, ^13^C-NMR, spectral comparison	[40]
Methyl gallate 3-*O*-β-d- (6′-*O*-galloyl)-glucopyranoside	Galloyl derivative	Methanol	FABMS, ^1^H-, ^13^C-NMR, TOCSY, DEPT, HETCOR, HMBC	[40]
Icariside B2	Terpene glucoside	Methanol	FABMS, ^1^H-, ^13^C-NMR, spectral comparison	[40]
3,3′,4-tri-*O*-methylellagic acid	Ellagic acid derivative	Methanol (aq.)	^1^H-NMR, UV–Vis, MS/MS	[15]
Gallic acid	Phenolic acid	Methanol (aq.)	^1^H-NMR, UV–Vis, MS/MS	[15]
3-*O*-Methylellagic acid	Ellagic acid derivative	Methanol (aq.)	^1^H-NMR, UV–Vis, MS/MS	[15]
Ellagic acid	Phenolic derivative	Methanol (aq.)	^1^H-NMR, UV–Vis, MS/MS	[15]
Methyl gallate	Phenolic acid derivative	Methanol (aq.)	^1^H-NMR, UV–Vis, MS/MS	[15]
*p*-Coumaric acid	Hydroxycinnamic acid	Methanol (aq.)	^1^H-NMR, UV–Vis, MS/MS	[15]
Valoneic acid dilactone methyl ester	Ellagic acid derivative	Methanol (aq.)	^1^H-NMR, UV–Vis, MS/MS	[15]
Quercetin 3-(2″-*O*-galloyl)- β-d-glucopyranoside	Flavonoid glycoside	Methanol (aq.)	^1^H-NMR, UV–Vis, MS/MS, acid hydrolysis	[15]
Oenothein B	Ellagitannin	Methanol (aq.)	^1^H-NMR, UV–Vis, MS/MS	[15]
Vicenin-1	Flavonoid *C*-glycoside	Methanol (aq.)	^1^H-NMR, UV–Vis, MS/MS	[15]
Isovitexin 2″-*O*-β-l-arabinoside	Flavonoid glycoside	Methanol (aq.)	MS/MS, acid hydrolysis, TLC sugar analysis	[15]
Isovitexin 2″-*O*-β-d-glucopyranoside	Flavonoid glycoside	Methanol (aq.)	MS/MS, acid hydrolysis, TLC sugar analysis	[15]
3-*O*-Methylellagic acid 4′-*O*-β-d-xylopyranoside	Ellagic acid glycoside	Methanol (aq.)	^1^H-NMR, UV–Vis, MS/MS	[15]
3-*O*-Methylellagic acid 4′-*O*-α-l-rhamnopyranoside	Ellagic acid glycoside	Methanol (aq.)	^1^H-NMR, UV–Vis, MS/MS	[15]
Apigenin 6,8-di-*C*-α-l- Arabinopyranoside	Flavonoid glycoside	Methanol (aq.)	^1^H-NMR, UV–Vis, MS/MS	[15]

* HRFABMS—High-Resolution Fast Atom Bombardment Mass Spectrometry; ^1^H-NMR—Proton Nuclear Magnetic Resonance Spectroscopy; ^13^C-NMR—Carbon-13 Nuclear Magnetic Resonance Spectroscopy; DEPT—Distortionless Enhancement by Polarization Transfer; COSY—Correlation Spectroscopy; HETCOR—Heteronuclear Correlation Spectroscopy; HMBC—Heteronuclear Multiple Bond Correlation Spectroscopy; TLC—Thin-Layer Chromatography; UV-Vis—Ultraviolet–Visible Spectroscopy; MS/MS—Tandem Mass Spectrometry; FABMS—Fast Atom Bombardment Mass Spectrometry; TOCSY—Total Correlation Spectroscopy.

## Data Availability

This article is a review of existing literature and does not report any new data; therefore, data sharing is not applicable.

## Abbreviations

The following abbreviations are used in this manuscript:

HPLC-DAD-MS^3^	High-Performance Liquid Chromatography coupled with Diode-Array Detection and Multistage Mass Spectrometry
HPLC-UV-ESI/MS	High-Performance Liquid Chromatography coupled with Ultraviolet Detection and Electrospray Ionization Mass Spectrometry
HPLC-DAD-MS/MS	High-Performance Liquid Chromatography coupled with Diode-Array Detection and Tandem Mass Spectrometry
PC	Paper Chromatography
2D-PC	Two-Dimensional Paper Chromatography
TLC	Thin-Layer Chromatography
UV	Ultraviolet Spectroscopy
HRFABMS	High-Resolution Fast Atom Bombardment Mass Spectrometry
^1^H-NMR	Proton Nuclear Magnetic Resonance Spectroscopy
^13^C-NMR	Carbon-13 Nuclear Magnetic Resonance Spectroscopy
COSY	Correlation Spectroscopy
HETCOR	Heteronuclear Correlation Spectroscopy
HMBC	Heteronuclear Multiple Bond Correlation Spectroscopy
UV–Vis	Ultraviolet–Visible Spectroscopy
MS/MS	Tandem Mass Spectrometry
FABMS	Fast Atom Bombardment Mass Spectrometry
TOCSY	Total Correlation Spectroscopy
GC-MS	Gas Chromatography–Mass Spectrometry
MW	Molecular Weight
NF-κB	Nuclear Factor kappa B
MAPK	Mitogen-Activated Protein Kinase
ICP–MS	Inductively Coupled Plasma Mass Spectrometry
DPPH	2,2-Diphenyl-1-picrylhydrazyl
SC_50_	Half maximal scavenging concentration
IC_50_	Half maximal inhibitory concentration

## References

[1] Tran N., Pham B., Le L. (2020). Bioactive compounds in anti-diabetic plants: From herbal medicine to modern drug discovery. Biology.

[2] Dar R.A., Shahnawaz M., Ahanger M.A., Majid I.U. (2023). Exploring the diverse bioactive compounds from medicinal plants: A review. J. Phytopharm..

[3] Wang Q., Ding L., Wang R., Liang Z. (2023). A review on the morphology, cultivation, identification, phytochemistry, and pharmacology of *Kitagawia praeruptora* (Dunn) Pimenov. Molecules.

[4] Shawky E.M., Elgindi M.R., Ibrahim H.A., Baky M.H. (2021). The potential and outgoing trends in traditional, phytochemical, economical, and ethnopharmacological importance of family Onagraceae: A comprehensive review. J. Ethnopharmacol..

[5] Kozhantayeva A., Iskakova Z., Ibrayeva M., Sapiyeva A., Arkharbekova M., Tashenov Y. (2025). Phytochemical insights and therapeutic potential of *Chamaenerion angustifolium* and *Chamaenerion latifolium*. Molecules.

[6] Xu Z., Deng M. (2017). Onagraceae. Identification and Control of Common Weeds, Volume 2.

[7] Overson R.P., Johnson M.G., Bechen L.L., Kinosian S.P., Douglas N.A., Fant J.B., Hoch P.C., Levin R.A., Moore M.J., Raguso R.A. (2023). A phylogeny of the evening primrose family (Onagraceae) using a target enrichment approach with 303 nuclear loci. BMC Ecol. Evol..

[8] Xie L., Wagner W.L., Ree R.H., Berry P.E., Wen J. (2009). Molecular phylogeny, divergence time estimates, and historical biogeography of *Circaea* (Onagraceae) in the Northern Hemisphere. Mol. Phylogenet. Evol..

[9] Nikzat S., Ghasemzadeh-Baraki S., Naghiloo S. (2021). The influence of environmental heterogeneity on the morphological and genetic diversity of *Circaea lutetiana* (Onagraceae) in Hyrcanian forests. An. Jard. Bot. Madr..

[10] Wagner W.L., Hoch P.C., Raven P.H. (2007). Revised classification of the Onagraceae. Syst. Bot. Monogr..

[11] Iskakova Z., Kozhantayeva A., Temirbekova A., Mukhtubayeva S., Bissenova G., Tekebayeva Z., Almagambetov K., Tashenov Y., Sarmurzina Z. (2025). Green synthesis of silver nanoparticles using *Circaea lutetiana* ethanolic extract: Phytochemical profiling, characterization, and antimicrobial evaluation. Int. J. Mol. Sci..

[12] Boufford D.E. (1982). The systematics and evolution of *Circaea* (Onagraceae). Ann. Mo. Bot. Gard..

[13] Granica S., Piwowarski J.P., Kiss A.K. (2013). Polyphenol composition of extract from aerial parts of *Circaea lutetiana* L. and its antioxidant and anti-inflammatory activity in vitro. Acta Biol. Cracov. Ser. Bot..

[14] Bussmann R.W., Paniagua-Zambrana N.Y., Khutsishvili M., Kikvidze Z., Müller L., Bussmann R.W., Paniagua-Zambrana N.Y., Kikvidze Z. (2025). *Circaea lutetiana* L. Onagraceae. Ethnobotany of the Caucasus.

[15] Granica S., Kiss A.K. (2013). Secondary metabolites from aerial parts of *Circaea lutetiana* L. Biochem. Syst. Ecol..

[16] Boufford D.E., Raven P.H., Averett J.E. (1978). Glycoflavones in *Circaea*. Biochem. Syst. Ecol..

[17] Averett J.E., Boufford D.E. (1985). The flavonoids and flavonoid systematics of *Circaea* (Circaeeae, Onagraceae). Syst. Bot..

[18] Seavey S.R., Boufford D.E. (1983). Observations of chromosomes in *Circaea* (Onagraceae). Am. J. Bot..

[19] Nguyen H.D., Do H.D.K., Vu M.T. (2024). Comparative genomics revealed new insights into the plastome evolution of *Ludwigia* (Onagraceae, Myrtales). Sci. Prog..

[20] Pavlov N.V. (1956). Flora of Kazakhstan.

[21] Petrishina N.N., Nikolenko V.V., Popova Z.V. (2018). Anatomical and morphological structure of vegetative organs of *Circaea lutetiana* L. Uchenye Zap. Krymskogo Fed. Univ. Im. V.I. Vernadskogo. Biologiya. Khimiya.

[22] Boufford D.E. (1982). The genus *Circaea* (Onagraceae) in Japan. Acta Phytotax. Geobot..

[23] Vakhrusheva L.P., Nurmambetova E.D. (2018). Age stages morphological criteria of *Circaea lutetiana* L. Acta Biol. Univ. Taur..

[24] Graae B.J., Ejrnæs R., Lang S.I., Meineri E., Ibarra P.T., Bruun H.H. (2004). The importance of recruitment limitation in forest plant species colonization: A seed sowing experiment. Flora.

[25] De Frenne P., Kolb A., Verheyen K., Brunet J., Chabrerie O., Decocq G., Diekmann M., Eriksson O., Heinken T., Hermy M. (2009). Unravelling the effects of temperature, latitude and local environment on the reproduction of forest herbs. Glob. Ecol. Biogeogr..

[26] Gorb E., Gorb S. (2002). Contact separation force of the fruit burrs in four plant species adapted to dispersal by mechanical interlocking. Plant Physiol. Biochem..

[27] El Zein H., Bou Dagher-Kharrat M. (2021). New records of vascular plants for the flora of Lebanon: A rare species rediscovered after seventy years, *Daphne pontica* L. (Thymelaeaceae), and three new occurrences, *Atropa bella-donna* L. (Solanaceae), *Circaea lutetiana* L. (Onagraceae), and *Euonymus latifolius* (L.) Mill. (Celastraceae). Check List.

[28] Godefroid S., Rucquoij S., Koedam N. (2005). To what extent do forest herbs recover after clearcutting in beech forest?. For. Ecol. Manag..

[29] Dimitrova M., Sulikovska I., Tsvetanova E., Djeliova V., Vasileva A., Ivanov I. (2025). Chemical composition and biological activity of extracts from the aerial parts of *Epilobium parviflorum* Schreb. Appl. Sci..

[30] Punt W., Rovers J., Hoen P.P. (2003). The Northwest European Pollen Flora, 67: Onagraceae. Rev. Palaeobot. Palynol..

[31] Koleckar V., Kubikova K., Rehakova Z., Kuca K., Jun D., Jahodar L., Opletal L. (2008). Condensed and hydrolysable tannins as antioxidants influencing the health. Mini Rev. Med. Chem..

[32] Cosme F., Aires A., Pinto T., Oliveira I., Vilela A., Gonçalves B. (2025). A comprehensive review of bioactive tannins in foods and beverages: Functional properties, health benefits, and sensory qualities. Molecules.

[33] Şabik A.E., Mohammed F.S., Sevindik M., Uysal I., Bal C. (2024). Gallic acid: Derivatives and biosynthesis, pharmacological and therapeutic effect, biological activity. Bull. Univ. Agric. Sci. Vet. Med. Cluj-Napoca Food Sci. Technol..

[34] Wianowska D., Olszowy-Tomczyk M. (2023). A concise profile of gallic acid—From its natural sources through biological properties and chemical methods of determination. Molecules.

[35] Yazar M., Sevindik M., Uysal I., Polat A.O. (2025). Effects of caffeic acid on human health: Pharmacological and therapeutic effects, biological activity and toxicity. Pharm. Chem. J..

[36] Silva T., Oliveira C., Borges F. (2014). Caffeic acid derivatives, analogs and applications: A patent review (2009–2013). Expert Opin. Ther. Pat..

[37] Yoshida T., Yoshimura M., Amakura Y. (2018). Chemical and biological significance of oenothein B and related ellagitannin oligomers with macrocyclic structure. Molecules.

[38] Lee E.J., Kim Y.S., Kim J.H., Woo K.W., Park Y.-H., Ha J.-H., Li W., Kim T.I., An B.K., Cho H.W. (2024). Uncovering the colorectal cancer immunotherapeutic potential: Evening primrose (*Oenothera biennis*) root extract and its active compound oenothein B targeting the PD-1/PD-L1 blockade. Phytomedicine.

[39] Kozhantayeva A.G., Rakhmadiyeva S.B. (2020). Research of polyphenolic compounds of *Circaea lutetiana* L. Chem. Bull. Kazakh Natl. Univ..

[40] Kim Y.C., Kingston D.G.I. (1996). A new caprylic alcohol glycoside from *Circaea lutetiana* ssp. canadensis. J. Nat. Prod..

[41] Granica S., Bazylko A., Kiss A.K. (2012). Determination of Macrocyclic Ellagitannin Oenothein B in Plant Materials by HPLC-DAD-MS: Method Development and Validation. Phytochem. Anal..

[42] Kozhantayeva A., Tashenov Y., Tosmaganbetova K., Tazhkenova G., Mashan T., Bazarkhankyzy A., Iskakova Z., Sapiyeva A., Gabbassova A. (2022). *Circaea lutetiana* (L) plant and its chemical composition. Rasayan J. Chem..

[43] Dima Ş., Dima C., Iordăchescu G. (2015). Encapsulation of Functional Lipophilic Food and Drug Biocomponents. Food Eng. Rev..

[44] Sundarraj S., Thangam R., Sreevani V., Kaveri K., Gunasekaran P., Achiraman S., Kannan S. (2012). β-Sitosterol from *Acacia nilotica* L. induces G2/M cell cycle arrest and apoptosis through c-Myc suppression in MCF-7 and A549 cells. J. Ethnopharmacol..

[45] Sirikhansaeng P., Tanee T., Sudmoon R., Chaveerach A. (2017). Major phytochemical as γ-sitosterol disclosing and toxicity testing in *Lagerstroemia* species. Evid.-Based Complement. Altern. Med..

[46] Viet T.D., Xuan T.D., Anh L.H. (2021). α-Amyrin and β-Amyrin Isolated from *Celastrus hindsii* Leaves and Their Antioxidant, Anti-Xanthine Oxidase, and Anti-Tyrosinase Potentials. Molecules.

[47] Nogueira A.O., Oliveira Y.I.S., Adjafre B.L., Amaral de Moraes M.E., Aragão G.F. (2019). Pharmacological Effects of the Isomeric Mixture of Alpha- and Beta-Amyrin from *Protium heptaphyllum*: A Literature Review. Fundam. Clin. Pharmacol..

[48] Velasco L., Goffman F.D. (1999). Tocopherol and fatty acid composition of twenty-five species of Onagraceae Juss. Bot. J. Linn. Soc..

[49] Matthäus B., Vosmann K., Pham L.Q., Aitzetmüller K. (2003). FA and tocopherol composition of Vietnamese oilseeds. J. Am. Oil Chem. Soc..

[50] Dolde D., Vlahakis C., Hazebroek J. (1999). Tocopherols in breeding lines and effects of planting location, fatty acid composition, and temperature during development. J. Am. Oil Chem. Soc..

[51] Kozhantayeva A., Rakhmadiyeva S., Ozek G. (2022). Evaluation of metal content of *Circaea lutetiana* (L) plant. J. Chem. Technol. Metall..

[52] Polezhaeva I.V., Polezhaeva N.I., Menyailo L.N. (2007). Amino acid and mineral compositions of the vegetative part of *Chamerion angustifolium*. Pharm. Chem. J..

[53] Elattar R.H., El-Deen A.K., Abdel Salam R.A., Hadad G.M., Khan A.A., Magdy G. (2026). Rapid Microwave-Assisted Synthesis of Silver Nanoparticles as a Luminescent Nanosensor for Baloxavir Marboxil Determination in Pharmaceuticals and Human Plasma: Comprehensive Assessment of the Method’s Sustainability. Luminescence.

[54] Yoshida T., Amakura Y., Yoshimura M. (2010). Structural features and biological properties of ellagitannins in some plant families of the order Myrtales. Int. J. Mol. Sci..

[55] Mihaylova R., Elincheva V., Momekov G., Simeonova R. (2025). Unlocking the therapeutic potential of ellagitannins: A comprehensive review of key representatives. Molecules.

[56] Tarawneh A.H. (2026). Cost-Effective Plant-Based Therapies for Diabetes-Hypertension Comorbidity: Evaluating Commercialized Medicinal Plants for ACE and α-Amylase Inhibitory Activity. Mor. J. Chem..

[57] Singh M., Mallick A.K., Banerjee M., Kumar R. (2016). Loss of outer membrane integrity in Gram-negative bacteria by silver nanoparticles loaded with *Camellia sinensis* leaf phytochemicals: Plausible mechanism of bacterial cell disintegration. Bull. Mater. Sci..

[58] Heleno S.A., Martins A., Queiroz M.J.R.P., Ferreira I.C.F.R. (2015). Bioactivity of phenolic acids: Metabolites versus parent compounds: A review. Food Chem..

[59] Gutiérrez-Grijalva E.P., Picos-Salas M.A., Leyva-López N., Criollo-Mendoza M.S., Vazquez-Olivo G., Heredia J.B. (2018). Flavonoids and Phenolic Acids from Oregano: Occurrence, Biological Activity and Health Benefits. Plants.

[60] Zhang Y., Cai P., Cheng G., Zhang Y. (2022). A Brief Review of Phenolic Compounds Identified from Plants: Their Extraction, Analysis, and Biological Activity. Nat. Prod. Commun..

[61] Sultana T., Ashrafi S., Das S., Hossain M.S., Ahsan M., Azam A.T.M.Z. (2024). Isolation of Flavonoids and a Triterpene Ester with Potential Bioactivity from *Ludwigia octovalvis* (Jacq.) P.H. Raven (Onagraceae) Leaves. Dhaka Univ. J. Pharm. Sci..

[62] Kandhare A.D., Bodhankar S.L., Mohan V., Thakurdesai P.A. (2016). Development and validation of HPLC method for vicenin-1 isolated from fenugreek seeds in rat plasma: Application to pharmacokinetic, tissue distribution and excretion studies. Pharm. Biol..

[63] Kandhare A.D., Bodhankar S.L., Mohan V., Thakurdesai P.A. (2016). Acute and repeated doses (28 days) oral toxicity study of vicenin-1, a flavonoid glycoside isolated from fenugreek seeds in laboratory mice. Regul. Toxicol. Pharmacol..

[64] Baruah T.J., Dutta S.P., Patar A.K. (2021). A preliminary in silico analysis of *Ocimum sanctum* flavonoids, orientin and vicenin-1, as potential drugs against SARS-CoV-2 infection. Int. J. Pharm. Sci. Res..

[65] Khuniad C., Nahar L., Talukdar A.D., Nath R., Ritchie K.J., Sarker S.D. (2025). Cancer chemopreventive potential of *Claoxylon longifolium* grown in Southern Thailand: A bioassay-guided isolation of vicenin 1 as the active compound and in silico studies on related C-glycosyl flavones. Molecules.

[66] Marrassini C., Davicino R., Acevedo C., Anesini C., Gorzalczany S., Ferraro G. (2011). Vicenin-2, a potential anti-inflammatory constituent of *Urtica circularis*. J. Nat. Prod..

[67] Islam M.N., Ishita I.J., Jung H.A., Choi J.S. (2014). Vicenin 2 isolated from *Artemisia capillaris* exhibited potent anti-glycation properties. Food Chem. Toxicol..

[68] Nagaprashantha L.D., Vatsyayan R., Singhal J., Fast S., Roby R., Awasthi S., Singhal S.S. (2011). Anti-cancer effects of novel flavonoid vicenin-2 as a single agent and in synergistic combination with docetaxel in prostate cancer. Biochem. Pharmacol..

[69] Huang G., Li S., Zhang Y., Zhou X., Chen W. (2020). Vicenin-2 is a novel inhibitor of STAT3 signaling pathway in human hepatocellular carcinoma. J. Funct. Foods.

[70] Ganesan K., Xu B. (2017). Molecular targets of vitexin and isovitexin in cancer therapy: A critical review. Ann. N. Y. Acad. Sci..

[71] Abdulai I.L., Kwofie S.K., Gbewonyo W.S., Boison D., Puplampu J.B., Adinortey M.B. (2021). Multitargeted effects of vitexin and isovitexin on diabetes mellitus and its complications. Sci. World J..

[72] Peng Y., Gan R.-Y., Li H.-B., Yang M., McClements D.J., Gao R., Sun Q. (2021). Absorption, metabolism, and bioactivity of vitexin: Recent advances in understanding the efficacy of an important nutraceutical. Crit. Rev. Food Sci. Nutr..

[73] Lu L., Deng Y., Li J., Feng X., Zou H. (2025). Molecular mechanisms of vitexin: An update on its anti-cancer functions. Int. J. Mol. Sci..

[74] Ali F., Rahul, Naz F., Jyoti S., Siddique Y.H. (2017). Health functionality of apigenin: A review. Int. J. Food Prop..

[75] Salehi B., Venditti A., Sharifi-Rad M., Kręgiel D., Sharifi-Rad J., Durazzo A., Lucarini M., Santini A., Souto E.B., Novellino E. (2019). The therapeutic potential of apigenin. Int. J. Mol. Sci..

[76] Karaoğlan E.S., Hancı H., Koca M., Kazaz C. (2023). Some bioactivities of isolated apigenin-7-O-glucoside and luteolin-7-O-glucoside. Appl. Sci..

[77] Gullón B., Lú-Chau T.A., Moreira M.T., Lema J.M., Eibes G. (2017). Rutin: A review on extraction, identification and purification methods, biological activities and approaches to enhance its bioavailability. Trends Food Sci. Technol..

[78] Al-Dhabi N.A., Arasu M.V., Park C.H., Park S.U. (2015). An up-to-date review of rutin and its biological and pharmacological activities. EXCLI J..

[79] Parveen Z., Deng Y., Saeed M.K., Dai R., Ahamad W., Yu Y.-H. (2007). Antiinflammatory and analgesic activities of *Thesium chinense* Turcz extracts and its major flavonoids, kaempferol and kaempferol-3-O-glucoside. Yakugaku Zasshi.

[80] Taiwo F.O., Oyedeji O., Osundahunsi M.T. (2019). Antimicrobial and antioxidant properties of kaempferol-3-O-glucoside and 1-(4-hydroxyphenyl)-3-phenylpropan-1-one isolated from the leaves of *Annona muricata* (Linn.). J. Pharm. Res. Int..

